# Do business records management affect business growth?

**DOI:** 10.1371/journal.pone.0264135

**Published:** 2022-03-10

**Authors:** Clement Mintah, Mohamed Gabir, Felicia Aloo, Elvis Kwame Ofori

**Affiliations:** 1 College of Economics and Management, Taiyuan University of Technology, Taiyuan, People’s Republic of China; 2 Faculty of Planning and Land Management, University for Development Studies, Tamale, Ghana; Universiti Pertahanan Nasional Malaysia, MALAYSIA

## Abstract

The implementation of a records management plan is an excellent approach to ensure small and medium-sized enterprises (SMEs) are sustained and continue to expand into huge or multi-national corporations. Maintaining records helps businesses in making better judgments and developing appropriate policies, resulting in enhanced effectiveness and efficiency. This will leverage means for tracking business progress and making appropriate decisions to expand the productive component of the economy. SMEs Business growth will help generate tax revenue for the government while also encouraging poverty reduction through tax transfers. We conducted a thorough investigation to determine the impact of each variable on business growth. For statistical analysis, a partial least squares structural equation modeling (PLS-SEM) methodology was applied. The results suggest that business records management and training have a positive indirect effect on business growth. However, the indirect effect of business records management policies insignificantly influences SMEs’ adoption of adequate record-keeping procedures, which harms business growth in Ghana. On the other hand, the total effect of the variables such as business records management training, business records management policies, and business records management positively impact business growth. Findings make a significant contribution to existing knowledge in the areas of record-keeping, management, and business growth. Business records management is an issue that requires more policy attention. This will business owners and managers strategically plan for new business directions based on data acquired. Proper record-keeping is necessary to satisfy end-users such as company directors, shareholders, external auditors, investors, creditors, and other interested parties. SMEs place a high value on business records management because of the impact it has on their long-term viability. The research outcomes provide a means for, and data on, business appraisal and management strategies.

## 1 Introduction

Small and medium-sized enterprises (SMEs) are critical to the economic success of most developed countries. Because complete and correct business records are vital for decision making, small-scale business operator units should ensure that they are retained [1]. This can be ensured by undergoing record-keeping training and recruiting informed and professional employees. SMEs employ approximately two-thirds of the country’s workforce, contribute to government income generation through income taxation, and offer household income through taxes, profits, and salaries, to name a few [2,3]. Therefore, there is a need to encourage SMEs to maintain proper records for the survival and growth of small businesses [4].

According to Prasad, Green [5] argues that increasing SMEs will help to expand the size of the economy’s directly productive sector; create government revenue; and, overall, support poverty reduction through tax transfers, job income, and business ownership. Apart from the tremendous and commendable contributions that these SMEs make to the economy, the industry still faces some obstacles that must be addressed if the sector’s potential is to be fully realized. The reliability of SME records keeps investors interested in investing and financial institutions interested in giving out loans to finance businesses [6].

In a study done by Tagoe, Anuwa-Amarh [6], SMEs have difficult times obtaining financial resources to support their operations because they lack sufficient financial records, which are required for loan access. To efficiently produce and supply services, entities must embrace good record maintenance in some manner. Maintaining good records is an important organizational resource that can serve as information and knowledge for the entity to become more functional, regardless of its format.

Many countries and researchers have regarded SMEs as the backbone of a country’s economic development [7–10]. Despite their contributions, these enterprises are confronted with numerous challenges in growing and managing their enterprises, which could lead to the collapse of these small and medium-sized firms, as suggested in [11]. As cited in Ezejiofor, Olise [12], 60 percent of SMEs still fail during the first five years of their establishment. As a result, SMEs must keep track of their operations to ensure their growth and long-term viability [13].

Further research by Nyabwanga and Ojera [14] shows that SMEs play an important role in reducing poverty and opening the door to national economic progress. Hence, a need to leverage means of supporting SMEs to maximize their potential.

Records management aims to provide precise and comprehensive information for operational decision-making in the organizational management process, according to Ngoepe [15]. Given the above, IRMT and WB [16,17] affirmed and stressed the necessity and need for commercial and public entities to keep proper records for the successful functioning and efficiency of organizations, no matter how small, as well as the format for records, keeping. According to IRMT and WB [16,17] maintaining appropriate records is important because it aids in the cataloging of government choices and activities, as well as those of other organizations, and thus serves as a baseline against which future decisions and activities can be assessed.

More specifically, Kusi, Yussif [18] asserted that issues including mismanagement and inadequate record-keeping procedures have been identified as severe management problems in the SME sector, resulting in the premature collapse of most entities. As a result, the focus of this study is on the relationship between business records management and its impact on the long-term viability of businesses.

## 2 Review of literature

This study made use of two dominant theories, namely the records life-cycle theory and the records continuum models, which are underpinning theoretical bases for records management and archival fields, to determine whether business records management can help in sustainable decision-making and business sustainability. According to McKemmish, Piggott [19] in the field of records management, other scholars have expressed interest in and acceptance of the records continuum model as an alternative to the records life-cycle model for accounting for records management approaches [20,21].

This viewpoint is shared by many archivists and records management experts. The records life-cycle theory is claimed to be the most comprehensive and integrated method of records management. The basic goal of a records management program, according to Mokhtar and Yusof [22] is to monitor records, regardless of kind or format, to guarantee that they transit efficiently and at a low cost through production, use, inactive storage, disposal, or permanent retention phases. Furthermore, Mokhtar and Yusof [22] feel that records management may be viewed as a valuable asset in the same way that cash, personnel, and tangible assets like land and buildings can. Both primary and secondary functions enhance accountability and reduce risks by assisting in the decision-making process. As a result, the records management idea can be incorporated into the overall business management strategy. Many archivists and records management specialists have endorsed this notion. The cycle idea is claimed to be the most comprehensive and integrated method for records management.

This explains why it is frequently used as a framework for managing public sector documents in Eastern and Southern Africa Ngulube [23]. However, research on private sector records management has gotten little attention. This will help strengthen policies and position records management as a management tool and a means for corporate sustainability. Meanwhile, the private sector has been hailed as a source of economic progress and a pioneer in the field of sustainable development [2]. Individual and company decisions and behaviors are frequently said to be based on theory. As a result, the record continuum theory gives theoretical support for the retention and management of commercial records.

In a more simple term, Dikopoulou and Mihiotis [24] defined records as information generated by an organization or individual and preserved by them to fulfill a legal duty or conduct business. Experts argue that record management entails applying systematic and scientific control to all recorded information that a company needs to run its operations sustainably. The concept of company records management is worth accepting for sustaining the failure of most SMEs through sound decision-making based on record generation, information, accessibility, authenticity, and utilization. This will aid in legal compliance, commercial considerations, and sustainability issues [9]. As a result, this concept has been adopted, modified, and expressed in a more business-oriented context as a means of ensuring the survival and sustainability of small and medium-sized firms.

### 2.1 The concept of SMEs

The primary importance of SMEs in the industrialization process and development of countries throughout the world, for sustaining growth and creating jobs in developing economies, cannot be overstated [25]. The vast significance of SMEs has been recognized by most business researchers. Given the significant role, they play in the economic sectors and countries’ progress, they have been proclaimed as an engine of growth and a development-driven instrument for the domestic economy [26]. SMEs, however, have different definitions in different countries. These are defined by the quantity of money invested, the rate of turnover, and the strength of the employee or team.

From a broader viewpoint, most SMEs in Canada, according to Adjei [27], use the employee threshold method as a criterion for classification. As a result, small businesses are defined as those with less than (100) employees, while medium-sized businesses are defined as those with fewer than (500) employees. In the case of Ghana, the National Board for Small Scale Industries (NBSSI) classified small enterprises based on the number of employees and fixed assets. Micro enterprises are defined as businesses with fewer than five people, whereas small-scale businesses have no more than nine employees and a maximum of 10 million Ghana Cedis in machine and equipment for their operations, according to the NBSSI [28]. Businesses with fewer than ten employees are considered and defined as small-scale firms, according to Ghana Statistical Service [29]. Medium and large-sized businesses are defined as companies with more than 10 employees. The employee threshold strategy of grouping organizations, Osei, Baah-Nuakoh [30] cited in Ibrahim and Musah [9] pre-engineered this conceptual idea. A micro firm, according to them, is a company with fewer than six employees, but a company with six to nine employees is considered tiny. They also designate a company as "small" if it employs 10 to 29 people. According to the referenced source, a total of 29 employees have been assigned to SMEs’ business classes [9]. As a result, the scope of this academic discussion includes the classification of SMEs as well as the methodology employed.

### 2.2 The position of the business records management concept in SMEs

Record keeping also referred to as documentation, is an organizational function that is responsible for the management of information throughout the development process of an organization, from generation to disposal. Document recognition, categorization, preservation, protection, extraction, monitoring, and disposal or permanent preservation are all part of this operation. Per the ISO 15489–1: 2001 benchmark, documents management is a field of management that is responsible for the efficient and systematic control of the creation, receipt, maintenance, use, and disposition of records, as well as the processes for capturing and maintaining evidence of and information about business activities and transactions in the form of records. According to the United Nations (UN), records are "information made, received, and preserved as evidence and information by an organization or person in the course of legal duties or the conduct of business by Australian Records Management Standards [31]. In the context of our work, what does it mean to keep records? It means that whenever you create or receive a document as part of your job that contains evidence of activity, decision, or transaction, you must keep it in compliance with UN retention schedules. That piece of paper becomes a record, and it must be stored in a safe place for as long as it is needed. Data collected on a specific subject or activity that is kept for future use is referred to as a record. Preserving records assists organizations in making better judgments and developing appropriate policies, resulting in enhanced effectiveness and efficiency [32].

In line with the above, Okoli [33] suggests that small and medium businesses may use a variety of record-keeping systems to achieve various purposes. The link between proper record keeping and small-scale business is a profitable one. Some of these services include assisting firms with operations control, business protection, and profit and loss monitoring. As a result, these allow business owners and managers to strategically plan for new business directions based on data acquired. As previously said, good record-keeping is necessary to satisfy end-users such as company directors, shareholders, external auditors, investors, creditors, and other interested parties. Good business records reflect how authentic, real, and fair business activities are, and they provide value to the company. More importantly, accurate records assist external auditors and revenue collectors and a major fault in financial administration originate from a failure to combine accounting and records management systems, resulting in inaccuracies and the loss of vital data [34].

The problems with the poor state of, SMEs’ records management systems are also stated explicitly as one of the main reasons why accounting standards will not be easily applied in developing countries [35]. When accounting systems are weakened as a result of inadequate record-keeping and administration, records may be unavailable for decision-making [15].

In light of the foregoing literature, it is prudent to keep and manage essential records properly throughout the RM life cycle to enable entrepreneurs to identify internal and external problems and work them out properly and effectively, as well as to use them as strategic planning tools for expanding their businesses.

The study concludes that any company that overlooks the need for effective record management techniques will most likely go out of business after the fiscal year and will always have issues with external auditors. Due to the commercial prospects it represents, most entrepreneurs keep records of prior events or activities and include them in their business planning and control. It is difficult for enterprises or organizations to avoid risk, as stated by [15]. What matters is being able to recognize and resolve issues that the company faces. Records management is essential for recognizing and analyzing hazards. This strategy aids in the formation and administration of a successful business. Management is the process of planning, controlling, organizing, staffing, leading, coordinating, and directing resources [36].

As a result, business management can be described as the act of utilizing available resources to achieve an organizational goal at the company level. The following records are kept in a business setting: cashbook, inventory book, a statement of financial position (balance sheet), a ledger or journal, a statement of revenue and expenditure, a statement of comprehensive income, a cash flow statement, and a cash budget are all examples of financial statements. Receipts and payments are all examples of books that are kept. These records are classified as non-human resources in business operations, but they play an important part in corporate planning, growth, and good decision-making processes.

Users must comprehend what management comprises to better grasp and appreciate that record management affects business. Human and non-human resources are leveraged into the management process to attain corporate goals [36]. Records keeping can be defined as one of the non-human organizational resources that, if properly maintained, will contribute to the growth and development of SMEs.

### 2.3 The need for SMEs to keep and manage records

According to Ernest [37] records management programs aids in improving the effectiveness of records as a management memory that manages the hours, equipment, and space given for records, and in simplifying intra-organizational and communication challenges by coordinating and protecting an institution’s records. According to the aforementioned literature analysis, information and data derived from a successful and efficient records management program help businesses plan effectively and make informed decisions, as well as preserve facts and statistics for future use. As a result, the enterprise’s organization and management will be more efficient and effective. Further research Amoako [38] confirms that the success and growth of a corporation are influenced by financial accounting systems. Other academics agreed that the success of the SME sector will be determined by the quality of financial accounting data used [39]. As a result, there is a link between the quality of accounting systems used in a commercial setting and the performance of the business.

The majority of SMEs have failed, owing to the weak accounting processes used by these businesses. Systems of accounting are critical in assessing a company’s profitability and growth [26,38,40]. It is critical for a system of accounting to be supported due to the following reasons; SMEs must employ an accounting system that allows them to determine the volume of sales, profits and losses, assets, and liabilities at any given time to achieve maximum business growth [41]. Records-keeping practices can be factored into business decision-making processes and thereby can be used as a strategic approach to enterprise growth and development. The failure of most enterprises in Ghana and Africa may be traced back to a lack of good record-keeping in our industry. Some business units in Ghana ask for and use daily reports and records for their operations.

Research conducted by Davis, Dunn [42] re-emphasized the need for adequate record-keeping in allowing state agencies to effectively plan their initiatives. Misappropriation of resources occurs in the majority of situations as a result of a lack of records or data when it is required by management. Customers can get in a bind with SMEs because of inaccurate records that make it difficult to completely explain and ascertain transactional concerns. Furthermore, managers can use accurate and readily available data to clarify particular concerns with consumers, clients, and the broader public. This aids in the avoidance of conflict and the establishment of solid customer relationships, as well as business growth and strategic business planning.

### 2.4 Challenging issues in records keeping and management

The underpinning issues in records keeping have been outlined by [43]. Inadequate records management policies, trouble managing electronic records, issues with standard compliance, poor staffing, and professionalism, and poor disposition and retention of records are all key issues. Further literature divulges; inadequate knowledge on record keeping, low educational levels, the cost for hiring professional accountants and owners attempt to keep records in their memory are also challenges that result in poor records-keeping among SMEs [44].

Owing to the above, Yussif, Kusi [26] posited that the main challenges of record-keeping in the SME sector can be said to be lack of knowledge, education or technical competence, inadequate logistical capacity as well as failure to recognize the essence of records, poor regulatory framework or standardization.

Furthermore, it is self-evident that preserving records is critical in the SME sector. However, a significant proportion of these SMEs have been found to keep no records at all [9]. There are numerous arguments for the current situation. More so, Kyobe, Molai [45] outline some of the main obstacles for managing records which are related to policy issues, compliance with laws and regulations, the nature of the records held and used, where they are located, accountability, and responsibility of users, and the type of data held and utilized. Despite other challenging issues being suffered by SMEs, record-keeping has been frequently cited as a key driver of success [46]. Proper record-keeping has been distinguished as a domineering factor influencing the performance of business entities.

Also, Mwangi, Ng’etich [47], further explained that inefficiency and lack of records have been undisputable factors in the collapse of many SMEs. Research by Ibrahim and Musah [9] stated that many SMEs failed because owners and managers were unable to make timely and important managerial choices due to a lack of proper records. Many people have mentioned the advantages of keeping records. Some people wonder why certain SME owners don’t keep track of their finances. It appears that the importance of record-keeping in corporate decision-making, growth, and development is often overlooked by entrepreneurs. Without a doubt, most business owners are unaware of the need for keeping records.

### 2.5 Theoretical and conceptual framework: Records management and business sustainability

To have a thorough understanding of business records management and SMEs’ long-term viability. The records continuum model Evans, McKemmish [48], and the decision-making theory [49]. The study was guided by a conceptual understanding of business sustainability and business records management as a framework-specific philosophy. The goal is to better understand the interactions between business records management and SMEs’ long-term viability (businesses).

**Fig 1:**
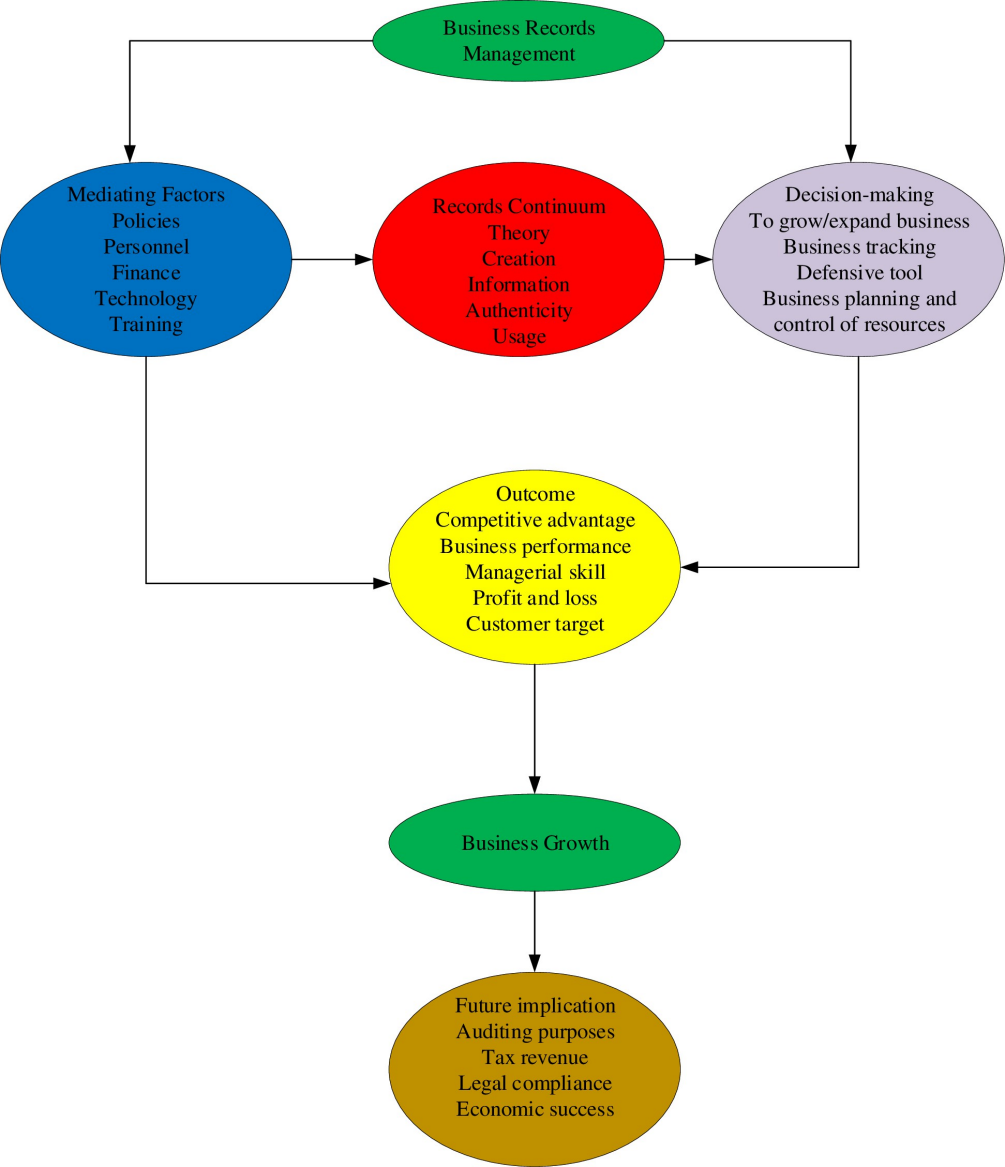
Conceptual framework.

In addition, we have presented the conceptual perspective of business records management and business development, and we have concluded by drawing a link between business records keeping entrepreneurial growth and development, and the records continuum theory, decision-making theory, and resource-based theory. However, all theories were explored, established, and linked. (See Fig 1). The concept of business records management is now positioned as a cross-edge approach to track business growth.

We have situated business records management as a strategic management tool for corporate sustainability and growth, complementing and serving as a critical engine for tracking regional, economic, and social development in Ghana. Given the important role that these SMEs play in economic development, the continuity of this action will place SMEs on a sustainable development scale, ensuring economic prosperity, since accurate and timely records of information help make good business management and operation decisions [1]. Hence, we accept the concept of business records management as a business management strategy and means of making successful decisions that lead to business growth as a good idea. We have empirically tested and established the connection between the dependent and the independent variables, the outcome of this research has been elucidated in the conceptual framework below.

More crucially, having acknowledged and echoed the key purpose of SMEs, and the government effort in promoting SMEs growth [2,7,50,51]. We complement government effort by researching and designing business records management concept to safeguard and sustain SMEs in Ghana and across the globe, emphasizing their complete implementation and use as a check for business growth, decision-making, and survival. The findings of this research aid the survival and expansion of small enterprises to grow into multinational companies. This scholarly study is an addition to the body of knowledge on records management techniques. The findings benefit all SMEs around the world, not just in the context of Ghana. This research will provide investors or stakeholders in businesses with the groundwork needed to develop and adopt rules and practices that will assist them in better managing business records. The results establish strong support for records management and business tracking and growth.

Geographically, the research covered the Eastern region of Ghana.

This is an area where SMEs owners faced various challenges in their recordkeeping process including lack of accounting knowledge and absence of specific guidelines for bookkeeping and accounting recordkeeping, fear of discouragement in case of a loss, inadequate education and training skills, and cost and time constraints [50]. This has contributed to the swift failure of most businesses in the area. The main objective was to find out the impact of business records management on business.

**Fig 2:**
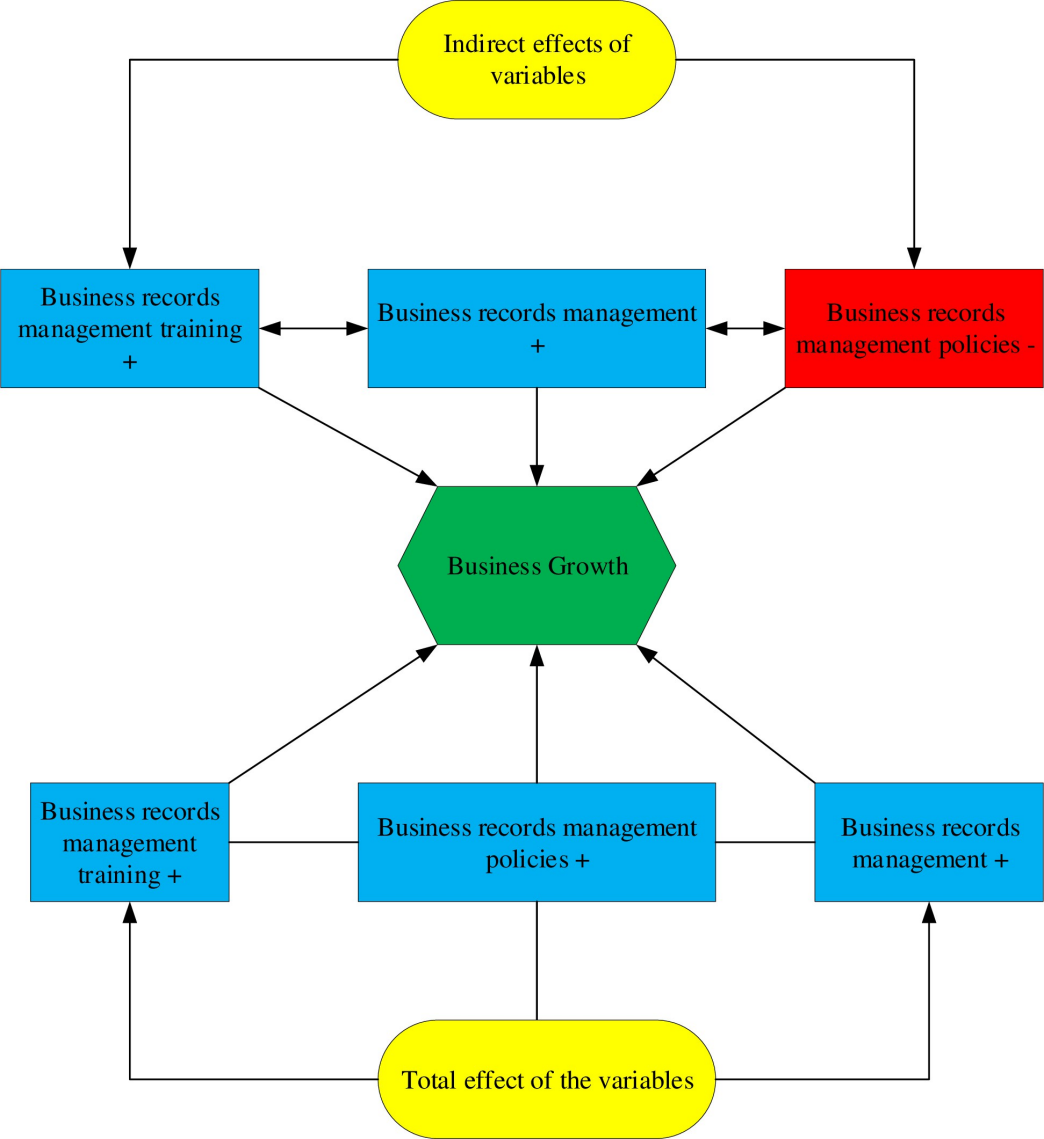
Graphical abstract.

Nonetheless, policymakers will have a starting point. The data reveal that company components that focus on spending have the greatest impact on business growth explanation. The study, on the other hand, SMEs ’ credit accessibility and records management compliance are critical variables that will drive economic progress. By embarking on this study, the importance of governments around the world using records management studies to guide policy changes has been stressed and echoed. After testing the hypotheses, the following observations were established:

**Hypothesis 1:** Business records management training is positively related to business records management policies.
**Hypothesis 2:** Business records management training is positively associated with business records management.
**Hypothesis 3:** Business records management training is insignificantly associated with business growth.
**Hypothesis 4:** Business records management policies are positively associated with business records management.
**Hypothesis 5:** Business records management policies are insignificantly associated with business growth.
**Hypothesis 6:** Business records management is positively associated with business growth (See Fig 2).

## 3 Modeling, methodological framework, and data

### 3.1 Model specification

We looked into the link between business records management and long-term business viability and business growth. This is an exploratory research work that used the survey approach. Using partial least squares structural equation modeling (PLS-SEM) methods, the investigation followed a well-established pattern. Because it can test theoretically supported linear and additive causal models, structural equation modeling (SEM) is a popular tool in marketing research. It is a second-generation multivariate data analysis method that is often employed in marketing research [52]. Marketers may use SEM to visually investigate the links that exist among factors of interest to prioritize resources to better serve their clients, a technique known as visual analysis. Because SEM may be used to analyses unobservable and difficult-to-measure latent variables1, it is an excellent tool for handling business research difficulties Bollen [53] and Jöreskog and Sörbom [54] employed structural equation modeling (SEM) for the first time in social sciences, serving as academic advisors for Wold [55] who founded the LISREL CB-SEM software package. Then, PLS-SEM was significantly improved by Ringle, Wende [56]. Hair, Ringle [57] explain that CB-SEM is used to evaluate the goodness of fit, which is concerned with minimizing the disagreement (differences) between the observed and calculated covariance matrices. Its use is recommended for testing and confirmation in cases when the preceding theory is robust or there is a compelling reason to do so. However, when doing CB-SEM, researchers or practitioners should make the assumption. The first is that the sample size of the data should be substantial, at least 200. Hair, Ringle [57] recommend the smallest sample size possible based on the model’s complexity and fundamental measurement model properties. According to Goodhue, Lewis [58], the sample size should not be the primary justification for using PLS-SEM because it lacks enough statistical power when sample sizes are small. They advocate PLS as a more powerful approach than CB-SEM when modest sample size is feasible. CB-SEM statistical software is available in AMOS, LISREL, MPLUS, and EQS, whereas PLS-SEM is available in SMART PLS. However, we opted to use smart pls as it suited our research objectives see [58–61].

We calculated descriptive statistics for variables in this article. The following is the general shape of our empirical model:

$$BG=\alpha_{0}+\beta_{1}RMP+\beta_{2}BRM+\beta_{3}BRMT+\varepsilon$$

Where;

BG = Business Growth

α = Trend/Constant

β = Coefficients

RMP = Records Management Policy

BRM = Business Records Management

BRMT = Business Records Management Training

ε = Error of term

#### 3.1.1. Ethical approval

Ethical approval was sought from the Taiyuan University of Technology Institutional Review Board (TYUTIRB/SEM/2021/89) and the National Board for Small-Scale Industries (NBSSI) in Ghana to conduct the study. A recording device was used to take oral consent from participants as indicated by the ethics committee. The purpose of the study and other details were disclosed to the authorities and participants. Participants were not financially induced or coerced to take part in the study. It was explained to them that their participation was voluntary.

### 3.2 Empirical results and discussions

The results are shown in Table 1 specifies the descriptive statistics of our respondents.

**Table 1:** Descriptive statistics for the sample. N = 329.

Variables		Frequency	Percent
Enterprise location (valid)	Koforidua	80	24
	Suhum	16	5
	Asamankese	34	10
	Nsawam	30	9
	Nkawkaw	44	13
	Oda	39	12
	Somanya	52	16
	Akropong	34	10
Gender (valid)	Female	121	37
	Male	208	63
Age (valid)	20–30	33	10
	31–40	52	16
	41–50	149	45
	51–60	77	23
	60+	18	5
Marital status (valid)	Single	191	58
	Married	48	15
	Divorced	30	9
	Widowed	60	18
Educational level (valid)	None	38	12
	Basic education	135	41
	Secondary education	126	38
	Tertiary	30	9
Type of businesses (valid)	Manufacturing	84	26
	Tailors and dressmakers	67	20
	Shoe-making	20	6
	Cassava processing	30	9
	Animal farming	36	11
	Hairstyling	44	13
	Carpentry	22	7
	Petty trading	26	8
Business registration (valid)	Yes	235	71
	No	94	29*
Place of business registration	District assembly	72	22
	National Board for Small Scale Industries	63	19
	Registrar General Department	98	30
	None	96	29*
Business role (valid)	Clerical	18	5
	Supervisory	90	27
	Managerial	221	67

Field survey, (2021).

From the data obtained, most of our respondents were males, which accounts for 63% whereas37% were females. This is an indication that most men are actively involved in doing business in Ghana. Again, 16% of our respondents were between the ages of 31–40, 45% between the ages of 41–50, 10% between the ages of 20–30, 23% between the ages of 51–60, and 5% are 60+ years respectively. Our survey made use of the working group of 15 years or older as specified by the International Labor Organization. A vast majority of our respondents were within the active population of the country. Hence, valid data were gathered from the respondents to form the basis and justification for the research.

Of the respondents, 9% had a tertiary level of education, followed by 38% with secondary education, 41% with basic education, and 12% representing respondents with no educational background. This is an indication that a higher percentage of the respondents who graduate from both basic and secondary schools in Ghana are actively involved in businesses. The findings from the descriptive statistics show that a higher percentage of our respondents are single which accounted for 58%, 15% are married, whereas 9% and 18% are divorced and widowed respectively. A high percentage of our respondents, therefore, were single. Perhaps this could be an indication that most SME operators are single, energetic, or have enough time to enough time to do business in Ghana without family stress and responsibilities. The SME operators are engaged in businesses such as manufacturing, tailoring & dress-making, hairstyling, animal farming, cassava processing, petty trading, carpentry, and shoemaking (26%, 20%, 13%, 11%, 9%, 8%, 7%, and 6%) respectively.

More importantly, the research divulges that a greater percentage (71%) of the SME operators have registered their businesses whereas 29% have not registered their businesses. This indicates that the government is losing 29% of its tax revenue if businesses are operating without licenses. A higher percentage (30%) have registered their business at Registrar General Department, 22% of the SMEs operators have registered with the District Assembly, 19% with the NBSSI, and as high as 29% have not registered with any of the above institutions. We, therefore, call on the agencies responsible for the task.

The survey results show that 67% of the SME operators, manage the business themselves, whereas 27% and 5% play supervisory and clerical roles respectively. Therefore, tax officials can contact SME operators who are operating informally within the business sector and accrue all tax revenues owed by them to the state for developmental projects.

### 3.3 Measurement of reliability and validity

Table 2 shows the Cronbach’s alpha and composite reliability values which were examined to determine validity and reliability of the scale for the research—the results for Cronbach’s alpha were higher than the recommended value of 0.7. This indicates that the scale merits the assessment of the research items. Specifying the respective loadings, the scale objects in the measurement model contributed significantly to this study, with a value between 0.766–0.976. This depicts excellent internal reliability see Table 1 Bagozzi and Yi [62]. Inferring from Fornell and Larcker [63], he argued that an average variance above 0.50 convergent validity is best achieved. The Average Variance Extracted (AVE) of all variables met the minimum requirement as the least was 0.591and the highest 0.881. Both reliability and internal consistency of all variables or constructs are also confirmed by rho_A and composite reliability (CR) values.

**Table 2:** Variable measurement and descriptive statistics.

	Variables	Factor loading	Cronbach’s Alpha	rho_A	C.R	AVE
1.B.R.M. Training	BRMT1	0.871	0.762	0.767	0.851	0.591
	BRMT2	0.798				
	BRMT3	0.890				
2.B.R.M. Policies	RMP1	0.976	0.954	0.956	0.967	0.881
	RMP2	0.973				
	RMP3	0.855				
	RMP4	0.945				
3.B.R.Mgt	BRM1	0.766	0.788	0.794	0.865	0.620
	BRM2	0.905				
	BRM3	0.927				
4.B.Growth	BG1	0.961	0.845	0.847	0.899	0.695
	BG3	0.928				
	BG4	0.956				

The result of the Fornell-Larcker criterion is shown in the lower triangle of Table 3. The square roots of the average variance extracted are shown in diagonal elements, with the findings of HTMT.85 marked with an asterisk in the upper triangle.

**Table 3:** Means, standard deviations, correlations, and discriminant analysis results tests.

Variables	Mean	SD	1	2	3	4
1.B.R.M. Training	4.258	0.793	0.854	0.521*	0.687*	0.355*
2.B.R.M. Policies	4.147	0.858	0.460	0.938	0.460*	0.224*
3.B.R.Mgt	4.089	0.830	0.570	0.415	0.869	0.446*
4.B.Growth	4.139	0.880	0.327	0.221	0.410	0.949

The results in Table 3 show that the tests are both valid and reliable. Heterotrait-Monotrait Ratio (HTMT) is a novel indicator for determining discriminant validity in variance-based measurement models like partial least squares (PLS). To be on the safer side, it is ideal to combine both testing methods to ascertain a reality despite Henseler’s demonstration regarding the latest method, HTMT, which outperforms the conventional Fornell-Larcker criterion [64]. The pairwise correlations between the other latent components were compared to the square roots of the AVE values using the Fornell-Larcker criterion. Discriminant validity is defined when the square root of the AVE is greater than the pairwise correlations for each construct of the measurement model, as was the case with the data reported in confirmed by rho_A and CR values.

Hypothesis result and consistent PLS. The partial least squares structural equation modeling is shown in Table 4. The offered hypotheses were evaluated using the PLS-SEM technique since we are confident of in-depth results as suggested by PLS-SEM [65]. If the investigation is about the evolution of an emergent classical model, Hair suggests using PLS-SEM. Given that the current study model is a Ghanaian adaption of Bang’s model, it is permissible to investigate Lin’s approach utilizing PLS-SEM instead of covariance-based structural equation modeling. In addition, rather than employing traditional PLS, this study used consistent PLS (PLSc) to assess our suggested model [61,66]. Smart-PLS 3 evaluates the metric model’s construct validity and tests the structural model’s parameters.

**Table 4:** Hypotheses testing results.

	Path Coefficient *β*	T Statistics	P Values	0.025%	0.975%	Remarks
H1. 1.B.R.M. Training -> 2.B.R.M. Policies	0.460	6.582	0.000	0.315	0.589	significant
H2. B.R.M. Training -> 3.B.R.Mgt	0.481	6.885	0.000	0.336	0.612	significant
H3. 1.B.R.M. Training -> 4.B.Growth	0.129	1.442	0.149	0.035	0.312	Not significant
H4. 2.B.R.M. Policies -> 3.B.R.Mgt	0.194	2.950	0.003	0.073	0.331	significant
H5. 2.B.R.M. Policies -> 4.B.Growth	0.026	0.368	0.713	0.109	0.170	Not significant
H6. 3.B.R.Mgt -> 4.B.Growth	0.326	3.904	0.000	0.157	0.480	significant
Residual Statistics	R	Q				
2.B.R.M. Policies	0.211	0.209				
3.B.R.Mgt	0.354	0.35				
4.B.Growth	0.182	0.174				

B.R.M. Training 2. B.R.M. Policies 3. B.R.Mgt 4. B. Growth.

### 3.4 Empirical results on the relationship between the construct items

To assess the suitability of the model, the normalized root means square residual is what we utilized (SRMR) [67], which was 0.041, an indication that the model is fit for the purpose and therefore credible, as it is less than the 0.08 parameter which should not be crossed [67,68]. We also reported R² values in Table 5 above. The R² value for the appropriate variable was used to evaluate each structural path; in general, the R² values were acceptable, so the research model’s explanatory power was all generally acceptable: since it falls within the required limit.

**Table 5:** Mediation analysis.

*Specific indirect effects*			
	*Indirect effects β*	T Statistics	P Values
H.1.1.B.R.M. Training -> 2.B.R.M. Policies -> 3.B.R.Mgt`	0.089	2.525	0.012
H.2.1.B.R.M. Training -> 2.B.R.3M. Policies -> 4.B.Growth	0.012	0.367	0.713
H3.1.B.R.M. Training -> 3.B.R.Mgt -> 4.B.Growth	0.157	3.452	0.001
H4.1.B.R.M. Training -> 2.B.R.M. Policies -> 3.B.R.Mgt -> 4.B.Growth	0.029	1.979	0.048
H.5.2.B.R.M. Policies -> 3.B.R.Mgt -> 4.B.Growth	0.063	2.251	0.024

***Author’s Investigation, 2021 reporting on specific indirect effects.

This finding is an indication that a mutual relationship exists between all the constructs within the research. Consequently, Q² was higher than 0. The results of Q² are a confirmation of the endogenous constructs’ predictive validity. Again, the Q² value greater than 0 specifies that the model is predictive.

#### Results of hypotheses (H1, H2, H3, H4, H5, and H6)

We had three distinctive results from the proposed test of the relationship between models, with two supported and vice versa.

The study’s findings reveal a positive and meaningful connection between business records management policies. (H1. 1.B.R.M. Training -> 2.B.R.M. Policies: *β =* 0.460 t = 6.582 p <0.000). We also tried to investigate the relationship between business records management training and business records management. The result shows that there exists a significant relationship (H2. B.R.M. Training -> 3.B.R.Mgt: *β =* 0.481 t = 6.885 p = 0.000). The relationship between business records management training and business growth as predicted is not statistically significant (H3.1.B.R.M. Training -> 4.B.Growth: *β =* 0.129 t = 1.442 p = 0.149).

Also, H4 evaluated the relationship between business records management policies and business records management. The relationship also reveals that the assertion is supported (H4. 2.B.R.M. Policies -> 3.B.R.Mgt: *β =* 0.194 t = 2.950 p = 0.003).

There exists a negative relationship between business records management and business growth (H5. 2.B.R.M. Policies -> 4.B.Growth: *β =* 0.026 t = 0.368 p = 0.713).

Again, the assessment of H6 (Business records management and business growth) established that there is a positive relationship between the construct items: H6. 3.B.R.Mgt -> 4.B.Growth *β =* 0.326 t = 3.904 p = 0.000).

We conducted mediation analysis in Table 5 to show how the role played by independent variables on independent variables and also to know their effects without a mediator looking at their total effects (without) mediation. All were significant except (H2 β = 0.012 t = 0.367 p = 0.713) where training had no indirect effect on records management and business growth.

However, the indirect effects of H1, H3, H4 and H5 were significant (H1: β = 0.089t = 2.525 p = 0.012), (H3: β = 0.157 t = 3.452 p = 0.001), (H4): β = 0.029 t = 1.979 p = 0.048, (H5: β = 0.063 t = 2.251 p = 0.024).

After conducting the mediation analysis, we further conducted another test in Table 6 shows the total effect of construct items to understand the overall or total role played by all interdependent variables on the independent variable. The result clearly shows that all were significant except (H5 β = 0.089 t = 1.248 p = 0.212) where business records management policies had no total effect on business growth. However, the total effects of H1, H2, H3, H4 and H6 were significant (H1: β = 0.46 t = 6.582 p = 0), (H2: β = 0.57 t = 9.02 p = 0), (H3: β = 0.327 t = 4.7362 p = 0), (H4: β = 0.194 t = 2.95 p = 0.003), (H6: β = 0.326 t = 3.904 p = 0).

**Table 6:** Total effect.

	Effect (*β)*	T Statistics	P Values
H1.1.B.R.M. Training -> 2.B.R.M. Policies	0.46	6.582	0
H2.1.B.R.M. Training -> 3.B.R.Mgt	0.57	9.02	0
H3.1.B.R.M. Training -> 4. B. Growth	0.327	4.736	0
H4.2.B.R.M. Policies -> 3.B.R.Mgt	0.194	2.95	0.003
H5.2.B.R.M. Policies -> 4.B.Growth	0.089	1.248	0.212
H6.3.B.R.Mgt -> 4. B. Growth	0.326	3.904	0

***Author’s Investigation, 2021 reported on specific indirect effects.

## 4 Discussion of result

Before the study, we established six hypotheses, to investigate the relationship between business records management and business growth in the Ghanaian context for SMEs.

To better understand the importance and mutual relationships that exist between business records management and the business growth of small and medium enterprises (SMEs) we placed the conceptualized business records management and positioned it’s as a business tracking and management strategy, means of competitive advantage by setting business records management as a defensive mechanism. Means of checking business growth, decision-making, and business survival.

The survival and growth of small-scale businesses can be promoted through these particular research findings because records management is vital to business management [69], having recognized the potential contribution of the SME sector to the growth of the Ghanaian economy in a wider perspective [9] due to the importance of SMEs in terms of job creation, government revenue generation, and poverty alleviation [70]. Hence, SMEs cannot be overlooked and allowed to fail within a few years of operation Ezejiofor, Olise [12] as a result of poor records management practices and continuous denial of access to credit facilities to finance their businesses [71]. The findings of this study make a significant contribution to existing information on record keeping, management, and business expansion. H1, H3, H4, and H5 have indirect effects on business growth, according to the findings. The P-values in the hypothetical scenarios are statistically significant. The results demonstrate that efficient company records management will have a positive impact on business growth. Second, the hypotheses’ combined effect (H1, H2, H3, H4, and H6) is statistically significant. This indicates that records management training has a favourable impact on the long-term viability of the company.

The test statistics have revealed that the mediating effect of business records management training through business records policies does not affect business growth because their values were insignificant. A follow-up test conducted on the total effect of construct items (business records management policies and business growth) also proved to be statistically insignificant. This shows that government policies on records keeping are unsuccessful in either supporting or enforcing the keeping and managing of records and sees records management as a field of interest in a business management approach that will be of benefit to economic growth. This research is in support of United Nations Sustainable Development Goal 8. Hence, a young entrepreneur should see business records management as a resource [22,72–74]. We, therefore, support the idea that entities must adapt to the proper maintenance of records to effectively deliver their services.

Our findings generally bring to bear that SMEs regard business records management due to its effect on business growth. Entrepreneurs must see business records management training, business records management policies, and business records management as considerable indicators for business growth and SMEs’ success. The application of these research findings could be a means of business appraisal and business management strategy, placing business at a competitive advantage, a defensive mechanism tool, and a tool for business decision-making, tracking business, performance, and survival.

The above test represents established proof that business records management improves the long-term viability of a company. We then support the idea that proper records management of SMEs and its effect on business growth is facilitated by the level of business records management training received and internally designed policies to keep business records. Hence, it is a business management concept that should be fully practiced by all SMEs; since business management and entrepreneurial success can be assured through such management processes to ensure the business sustenance, expansion, and growth of SMEs into multi-national companies which can enjoy economic prosperity in the future.

In this research, we, therefore, accept hypotheses (H1, H2, H4, and H6) due to their positive impact on business growth. We however reject hypotheses (H3 and H5) because policies do not significantly affect business growth, unless they are mediated through business records management training, business records management policies, and business records management to realize the total effect on business growth in this case.

## 5 Managerial implications and conclusion

Ensuring proper business records management within SMEs will leverage or aid policymakers, government officials, business managers, and young entrepreneurs to marshal or identify a laid down procedure and a means of business appraisal, management strategy, tax/audit, legal compliance, competitive advantage, a defensive mechanism tool, and business decision-making, tracking business profit and loss, and as a means to further plan, control, and direct business resources to target customers in Ghana.

In a broader sense, the records continuum theory ensures that records should be created, and become information and that information should be accessible, authentic, and can be used as when needed therefore supporting since its significance has been established in business sustainability. Hence, business records management is an influential variable and a driving indicator for business sustainability, having assessed its theoretical importance, testing of hypotheses, and practice. According to the survey results, business records management training, setting business records management policies and proper records management positively affect business growth. This will help business actors such as the government and, business owners/managers to make a decision based on business information available to either innovate or come up with new business plans to suit the taste and preferences of the changing business environment. This research is a call for policy concern for formal and informal businesses (SMEs) to position it as a daily routine and practiced within business frontiers.

In conclusion, the availability of business records and management of these will eventually help government officials to know of specific businesses that pay and invade taxes, to establish reasons for business failures and further direct policies and actions towards economic success. Finally, in literature, other variables such as personnel, finance, and technology are necessary factors that need to be considered in the quest to ensure business records management and its impact on business growth.

### 5.1 Limitations and future research

Our variables or construct items of analysis had some shortcomings, such as the possibility of some research questions not being answered due to the coronavirus disease COVID-19 pandemic which poses restrictions on collecting data from some areas, time limitations, and financial constraints in meeting the transportation cost of data collection. However, the researcher tried everything possible to engage respondents to elicit responses for the study. The research established strong policy support for business records management as a prerequisite and requirement for ensuring business growth due to its statistically significant relationship with other explanatory variables. But more importantly, further research can be conducted in other parts of Ghana by combining other variables such as personnel, finance, and technology which were not included in our hypotheses, these can also be tested to ascertain their significance on business growth.

### 5.2 Innovation of the paper

The study aimed at advocating and echoing strong business records keeping and management policy on the plausibility of business growth as a means of marshaling or identifying means of tracking business progress, a means of business appraisal, a means to develop a further plan, control, and direct business resources based on available business records to further position the business at a competitive advantage over other businesses within the same business environment. Having established this concept, it will eventually serve as a defensive mechanism tool, path for business decision-making and a means to target customers or consumers in Ghana. This will help maximize productivity and reduce the failure of most businesses as a result of improper records management practices. Hence, our findings are in support of the concept of business record-keeping and management and encourage it’s being fully practiced. After carefully assessing its importance in the business domain and its economic benefit.

At present, no such specific work has been done in Ghana to the extent of developing a conceptual framework for its adoption. This propels our interest in this area of research to investigate and develop such a concept as a practical means to manage and bring innovations into business management. Since records management is a field of management science, it is an area that needs attention and policy formation by focusing on its impact on business growth.

## Supporting information

S1 File (DOCX)
